# Safe prescribing in cognitively vulnerable patients: the use of the anticholinergic effect on cognition (AEC) tool in older adult mental health services

**DOI:** 10.1192/bjb.2019.43

**Published:** 2020-02

**Authors:** Delia Bishara, Charlotte Scott, Robert Stewart, David Taylor, Daniel Harwood, David Codling, Christine Banwell, Justin Sauer

**Affiliations:** 1 South London & Maudsley NHS Foundation Trust; 2 King's College London

**Keywords:** Dementia, anticholinergic burden, AEC scale

## Abstract

**Aims and method:**

Medication with anticholinergic action is associated with potentially serious adverse effects in older people. We present an evaluation of a novel anticholinergic burden scale introduced into routine practice in older adult services in the South London and Maudsley (SLaM) NHS Foundation Trust. Our aim was to assess whether this tool improved the accurate identification of anticholinergic medication and guided safer prescribing in cognitively vulnerable older people.

**Results:**

The introduction of the anticholinergic effect on cognition (AEC) tool into clinical practice led to an increase in the identification and reporting to general practitioners of anticholinergic medication from 11 to 85% of cases (*P* = 0.0015).

**Clinical implications:**

Application of the AEC tool led to improved detection of anticholinergic medication and advice to primary care on when a medication review is necessary. This is an important step towards improving the safety of prescribing in this patient group.

Medicines with an anticholinergic effect are associated with an increased risk of cognitive decline, dementia and death in older adults.^1–4^ Anticholinergic medication has also been shown to oppose the action of acetylcholinesterase inhibitors and therefore their clinical efficacy.^5,6^

National Institute for Health and Care Excellence guidance on dementia^7^ emphasises the importance of assessing anticholinergic burden in cognitively vulnerable older people using an appropriate assessment tool. It recommends that when assessing people for cognitive impairment or dementia, a medication review should be conducted to identify drugs that may cause cognitive impairment. An NHS England dementia diagnosis and management resource for general practitioners (GPs) recommends that where possible, drugs with a strong anticholinergic effect should be stopped or substituted for drugs with less anticholinergic activity.^8^

A number of anticholinergic scales already exist and have been developed in order to classify drugs according to their anticholinergic risk. These were generally based on the subjective experience of assessors and are usually dependent on the drug's general potency to muscarinic receptors.^9,10^ A classification system was developed by Bishara *et al*^11^ to identify specifically the anticholinergic effect on cognition (AEC) for medications commonly used in the elderly. The development of the AEC scale was evidence based and used transparent systematic methodology to evaluate a large number of medications, while taking into account not only the drug's affinity for muscarinic receptors but also its potential to cross the blood–brain barrier and previous reports on its influence on cognitive impairment.

This tool is designed to be used in healthcare settings to support clinicians to identify and manage anticholinergic medication for patients with cognitive impairment. The AEC tool classifies medication according to a ‘traffic light’ system, giving drugs an individual score of 0, 1, 2 or 3. A score of 0 means the medication has no anticholinergic effect on cognition, and a score of 3 means it has the most effect. The individual scores of all the medications that a patient is taking are then added together to give a total AEC score. It is recommended that those with cognitive impairment who are on medication with an *individual* AEC score of 2 or more, or have a *total* AEC score of 3 or more, have a medication review so that the offending drugs can be withdrawn or switched to a safer alternative. The aim is to reduce the total AEC score to the lowest possible value.

## Aim

Our aim was to determine whether the introduction of the AEC tool into routine practice in older adult services would improve the identification of medicines with a significant anticholinergic effect on cognition and lead to appropriate advice given to the patient's GP on which drugs to review. It is hoped that such an intervention would result in an overall reduction in anticholinergic burden in patients and thus improve prescribing safety. We introduced the AEC scale into three older adult services across South London and Maudsley (SLaM) NHS Foundation Trust to assess its role in the identification of anticholinergic drugs and subsequent advice given to primary care regarding medication reviews.

## Method

### Current practice at the time of the study

New patients referred to the Southwark and Lambeth Memory Service are assessed by one member of the multidisciplinary team (MDT). The MDT consists of registered mental health nurses, clinical support workers, clinical psychologists, social workers, psychiatry trainees and a consultant psychiatrist. Assessments are completed using a document template which forms the basis of a report that is sent to the GP and uploaded onto the patient's electronic health records. This includes information in relation to the clinical history and examination, diagnosis, the patient's management plan and recommendations to the GP. All new assessments are discussed in the weekly MDT meeting. Prior to the introduction of the AEC tool, there was no formal template for assessing the anticholinergic burden of patients' medications.

Before introducing the intervention, retrospective baseline systematic sampling was used to identify 70 new patients assessed in the Southwark and Lambeth Memory Service over a 3 month period from January 2016. Data were collected by two psychiatry trainee doctors and included information retrieved from the patient's electronic patient record, including the patient's diagnosis, their full medication list and AEC scores for individual drugs. From this, the total AEC score was calculated. Information was also gathered on whether there was documentation that the anticholinergic medications and their potential anticholinergic burden had been identified, and whether this was communicated to the GP with appropriate advice.

### The intervention

In October 2016, the AEC scale was introduced into three separate older adult services across SLaM. SLaM is one of Europe's largest healthcare providers for mental health and dementia and serves a local population of 1.3 million people. The following teams were included in the study: Southwark and Lambeth Memory Service, Lewisham Memory Service and Lewisham Care Home Intervention Team (CHIT). A CHIT was included as it was considered that these teams are ideally placed to review anticholinergic medication in patients with dementia. A training session was provided to educate all staff conducting new assessments for patients on how to use the AEC scale, along with guidance on how to communicate high-risk drugs to the GP, while providing appropriate advice on which drugs to review. The assessment document template was updated to include, under the management plan, a standard communication to the GP informing them about the quality improvement project and a section to document the patients' individual drug and total AEC scores. Depending on the AEC scores, appropriate advice on whether a medication review was deemed necessary or not then followed. If the individual AEC score for a drug was 2 or more, or if the total AEC score was 3 or more, the advice to the GP was ‘we recommend that the following drugs be reviewed so as to reduce the anticholinergic burden on cognition’. If the total AEC score was under 3 and there were no individual drugs with an AEC score of 2 or more, then the advice was ‘there is no need for further review’. This advice is based on similar scales, such as the anticholinergic cognitive burden scale, which has been used to show that this scoring system reflects the potential clinical implications.^3^

Following the intervention, systematic sampling identified 98 patients newly assessed in the Southwark and Lambeth Memory Service from 1 September 2016 to 31 December 2016. Data were collected by a psychiatry trainee doctor, a consultant psychiatrist and a consultant pharmacist. The admission documents for the patients identified were again examined to determine whether anticholinergic medication had been correctly identified and communicated to the GP. Post-intervention data were also collected from the Lewisham Memory Service and CHIT by the designated pharmacist, so as to assess whether the intervention could easily be incorporated into the practice of other teams as well.

## Results

In the Southwark and Lambeth Memory Service, pre-intervention/baseline results showed that 20 patients (29%) were on anticholinergic medication. Similarly, post-intervention results showed that 30 patients (30%) were on medication with an anticholinergic effect on cognition. Eleven patients from the pre-intervention sample (16%) and 13 patients from the post-intervention sample (13%) were on medications with an individual AEC score of 2 or more (Table 1).

**Table 1:** Identification of medications with an individual AEC score of 2 or more (SLMS)

	Pre-intervention *n* = 70 (%)	Post-intervention *n* = 98 (%)
Patients taking medication with an individual AEC score of 2 or more	11 (16)	13 (13)
Documentation of identification of drugs with an individual AEC score of 2 or more, *n* (%)	4 (36)	11 (85)

AEC, anticholinergic effect on cognition; SLMS, Southwark and Lambeth Memory Service.

Clinicians identified four out of the 11 patients (36%) from the pre-intervention sample, i.e. those prescribed drugs with an individual AEC score of 2 or more (Table 1). The identification of an anticholinergic burden (score of 2 or more on the AEC) improved to 11 out of the 13 (85%) following the study intervention.

**Fig. 1:**
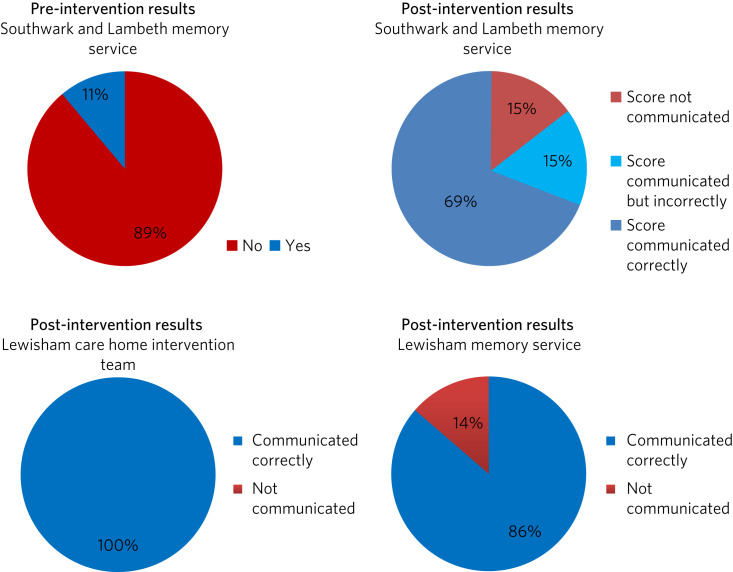
Pie charts showing the improvement of identification and communication to the GP of patients on medication with a total anticholinergic burden of 3 or more.

Nine patients (13%) from the pre-intervention sample and 13 patients (13%) from the post-intervention sample had a total AEC score of 3 or more (Table 2). Only one of the 9 patients in the pre-intervention sample with a total AEC score of three or more (11%) had this identified and communicated to the GP. Identification and communication of total AEC scores of 3 or more improved with the intervention. Eleven of 13 post-intervention patients (85%) had their total AEC score of 3 or more identified and communicated to the GP (see Fig. 1). A χ^2^-test (Fisher's exact test) showed that this improvement was statically significant (*P* = 0.0015).

**Table 2:** Identification of medications with total AEC score of 3 or more and communication to GP (SLMS)

	Pre-intervention *n* = 70 (%)	Post-intervention *n* = 98 (%)
Patients with total AEC score of 3 or more	9 (13)	13 (13)
Documentation that patients with total AEC score of 3 or more were identified and score communicated to GP		
Anticholinergic burden communicated to the GP	1 (11)	11 (85)
Total AEC score communicated correctly	8 (89)	9 (69)
Total AEC score communicated but calculated incorrectly		2 (15)
Total AEC score (of 3 or more) not identified nor communicated		2 (15)

AEC, anticholinergic effect on cognition; GP, general practitioner; SLMS, Southwark and Lambeth Memory Service.

However, of the patients whose AEC scores were communicated to the GP, two patients (15%) had their total scores calculated incorrectly (errors in the AEC scores and calculations were also noted for patients where the total score was less than 3). Reasons for the miscalculations included medications not being correctly identified owing to having multiple names (e.g. dosulepin's score was considered not known; however, it was listed on the AEC scale as having a score of 3 under the name dothiepin), medications' AEC score not being correctly identified on the scale and medications being included twice in the calculation. For the remaining two patients (15%) with a total AEC score of 3 or more, this was not identified and not communicated to the GP for reasons unknown.

Post-intervention data collected from the Lewisham teams showed that 40% of patients from the CHIT and 29% of patients from the memory service were on anticholinergic medication. The high rate of identification of anticholinergic medication with the use of the AEC tool was also reflected in these teams, using the methods described above. Post-intervention results showed that the anticholinergic burden had been communicated to the GP for 100% (7 out of 7) of patients with a total AEC score of 3 or more in the CHIT team and for 86% (19 out of 22) of patients in the memory service.

## Discussion

This study provides evidence that without a structured tool, the identification of medications with an anticholinergic effect on cognition and subsequent communication with advice to primary care is poor. With the introduction of the AEC tool and inclusion of an anticholinergic burden section in the assessment document, identification and communication with appropriate advice to the GP regarding these drugs greatly improved. This study suggests that with a brief training session using the AEC tool, identification of medications with clinically significant high AEC scores are identified and communicated to the GP in 85–100% of cases. This is a significant improvement from pre-intervention results showing that in only 11–36% of cases was identification of anticholinergic burden noted and communicated to the GP.

Where a psychotropic medication is causing a cognitive burden, this can represent a challenge, particularly if a patient has had a stable mental state over a sustained period of time. The mental health team may be able to suggest an alternative treatment in such situations and consider the risks versus benefits of switching.

## Limitations

Our intervention greatly improved the identification of and communication to GPs regarding anticholinergic medications. However, at times, the AEC calculations provided to the GP were incorrect. Miscalculations of the AEC score were secondary to human error (drugs being included twice in the calculation or confusion regarding drug names leading to misidentification of the AEC score). These findings alerted us to the importance of having an electronic version of the AEC scale, so as to eliminate such errors arising from misidentification of drugs from the list or simple calculation errors when adding up total scores.

## Developments

**Fig. 2:**
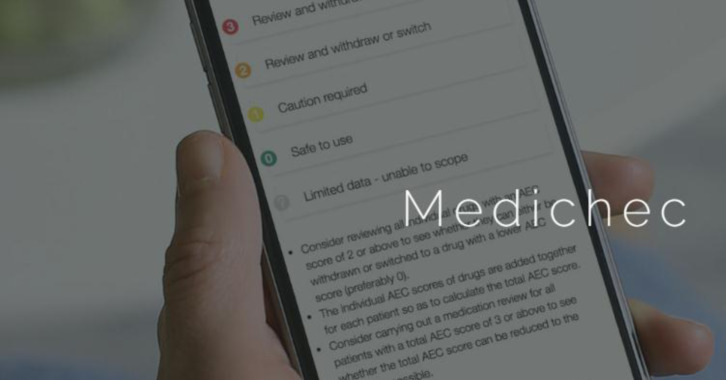
Image of Medichec app.

The results of this quality improvement programme led to the development of Medichec, a web-based and application version of the AEC tool. This allows practitioners to easily check their patient's medication, and the cumulative anticholinergic burden score is automatically calculated. The system will identify a drug and its score by any of the names listed in the British National Formulary. It alerts the practitioner as to which drugs need to be reviewed and gives advice on how to interpret the score in order for them to be communicated to the GP. Medichec only uses generic names and prompts the user when a trade name is used. It contains over 2000 medications and is now being used across SLaM's older adult services and in other areas of the UK. The AEC tool is available online as a free resource at http://www.medichec.com. An Android application (see Fig. 2) is also available, with plans for an Apple application to follow shortly. Plans are also underway to see whether Medichec can be incorporated into GP health record systems so as to improve accessibility and safer prescribing for cognitively vulnerable patients in primary care.

## About the authors

**Delia Bishara** is a consultant pharmacist, Mental Health of Older Adults (MHOA) and Dementia, SLaM, London, UK. **Charlotte Scott** is a speciality registrar at SLaM, London, UK. **Robert Stewart** is Professor of Psychiatric Epidemiology & Clinical Informatics at the Biomedical Research Centre, SLaM and King's College London, UK. **David Taylor** is Director of Pharmacy and Pathology and Head of the Pharmaceutical Sciences Clinical Academic Group, King's Health Partners, SLaM, London, UK. **Daniel Harwood** is a consultant psychiatrist and Clinical Director of MHOA and Dementia Clinical Academic Group, SLaM, London, UK. **David Codling** is a senior registrar at SLaM, London, UK. **Christine Banwell** is a pharmacist at SLaM, London, UK. **Justin Sauer** is a consultant psychiatrist and Clinical Lead for Memory Services at SLaM, London, UK.

## References

[1] Carriere I, Fourrier-Reglat A, Dartigues JF, Rouaud O, Pasquier F, Ritchie K, Drugs with anticholinergic properties, cognitive decline, and dementia in an elderly general population: the 3-city study. Arch Intern Med 2009; 169: 1317–24. 19636034 10.1001/archinternmed.2009.229 PMC2933398

[2] Jessen F, Kaduszkiewicz H, Daerr M, Bickel H, Pentzek M, Riedel-Heller S, Anticholinergic drug use and risk for dementia: target for dementia prevention. Eur Arch Psychiatry Clin Neurosci 2010; 260(Suppl 2): S111–5. 20960005 10.1007/s00406-010-0156-4

[3] Fox C, Richardson K, Maidment ID, Savva GM, Matthews FE, Smithard D, Anticholinergic medication use and cognitive impairment in the older population: the medical research council cognitive function and ageing study. J Am Geriatr Soc 2011; 59: 1477–83. 21707557 10.1111/j.1532-5415.2011.03491.x

[4] Gray SL, Anderson ML, Dublin S, Hanlon JT, Hubbard R, Walker R, Cumulative use of strong anticholinergics and incident dementia: a prospective cohort study. JAMA Internal Med 2015; 175: 401–7. 25621434 10.1001/jamainternmed.2014.7663 PMC4358759

[5] Lu CJ, Tune LE. Chronic exposure to anticholinergic medications adversely affects the course of Alzheimer disease. Am J Geriatr Psychiatry 2003; 11: 458–61. 12837675

[6] Sink KM, Thomas J III, Xu H, Craig B, Kritchevsky S, Sands LP. Dual use of bladder anticholinergics and cholinesterase inhibitors: long-term functional and cognitive outcomes. J Am Geriatr Soc 2008; 56: 847–53. 18384584 10.1111/j.1532-5415.2008.01681.x PMC4646065

[7] National Institute for Health and Care Excellence. Dementia: Assessment, Management and Support for People Living With Dementia and Their Carers. NICE, 2018 (https://www.nice.org.uk/guidance/ng97/resources/dementia-assessment-management-and-support-for-people-living-with-dementia-and-their-carers-pdf-1837760199109). 30011160

[8] NHS England. Dementia Diagnosis and Management. A Brief Pragmatic Resource for General Practitioners. NHS England, 2015 (https://www.england.nhs.uk/wp-content/uploads/2015/01/dementia-diag-mng-ab-pt.pdf).

[9] Boustani M, Campbell N, Munger S, Maidment I, Fox C. Impact of anticholinergics on the aging brain: a review and practical application. Aging Health 2008; 4: 311–20.

[10] Duran CE, Azermai M, Vander Stichele RH. Systematic review of anticholinergic risk scales in older adults. Eur J Clin Pharmacol 2013; 69: 1485–96. 23529548 10.1007/s00228-013-1499-3

[11] Bishara D, Harwood D, Sauer J, Taylor D. Anticholinergic effect on cognition (AEC) of drugs commonly used in older people. Int J Geriatr Psychiatry 2017; 32(6): 650–6. 27280553 10.1002/gps.4507

